# Supplementary material for: Semi‐automated workflow for high‐throughput Agrobacterium‐mediated plant transformation
Source: Plant J. 2025 Apr 12;122(1):e70118. doi: 10.1111/tpj.70118 (PMC11993085; doi:10.1111/tpj.70118)
Supplement: Supplementary file 1 — Appendix S1. File S1. Protocol designer file for Agrobacterium competent cells dispensation in 96‐well plates. File S2. Protocol designer file for Agrobacterium transformation automation for 24 samples. File S3. Protocol designer file for Agrobacterium transformation automation for 96 samples transformation. File S4. Protocol designer file for plating Agrobacterium transformations. File S5. Jupyter Notebook protocol for Agrobacterium transformation. File S6. Jupyter Notebook protocol for plating Agrobacterium transformations. [file TPJ-122-0-s006.zip › Supplemental Document 1.pdf]

Supplemental Document 1.

Protocol for *Agrobacterium* transformation by freeze-thaw method

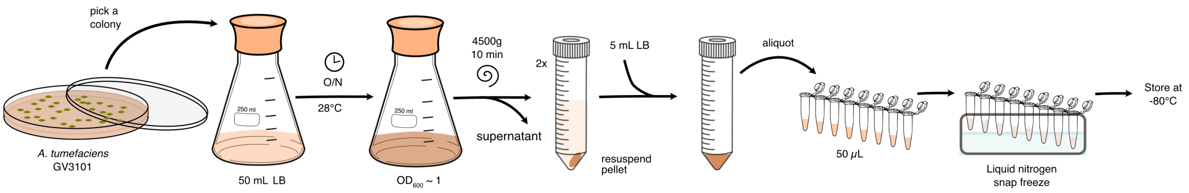

Figure 1. ***Preparation of freeze-thaw competent Agrobacterium cells.***

1) Inoculate two 250 mL flasks with 50 mL of LB media with *Agrobacterium tumefaciens* strain GV3101 and grow overnight at 28°C with shaking. These should reach an optical density at 600nm of approximately 1.

2) Transfer and split the cell suspension into two Falcon tubes. Pellet the cells at 4500 g for 10 min at room temperature.

3) Aliquot 50 μL of cells in sterile 200 μL PCR tubes for direct use, or store as 200μL aliquots in 1.5 mL Eppendorf tubes.

4) Snap freeze in liquid nitrogen and store at -80°C for future transformations.

Note: Agrobacterium competent cells can be used immediately without storing at -80 °C.

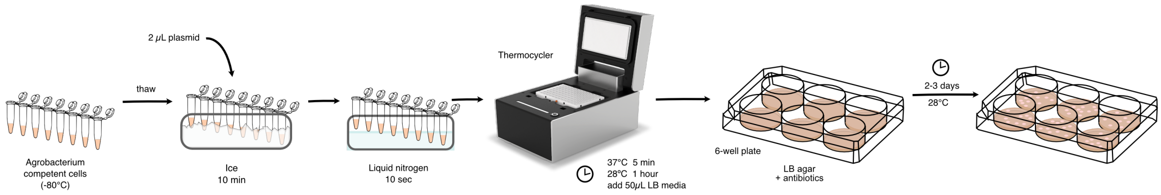

Figure 2. ***Freeze-thaw transformation of Agrobacterium cells.***

5) Thaw frozen competent cells for 10 min on ice.
6) Use 50 μL of competent cells per transformation (may need aliquoting if stocks are stored in 1.5 mL Eppendorf tubes) in 200μL PCR tubes or a 96 well microplate.
7) Add 2 μL of a plant transformation vector as plasmid DNA from a miniprep extract (~100 ng/μL). Mix by gently flicking.

Note: positive (control plasmid) and negative (water) controls can be included.

8) Flash-freeze the tubes in liquid nitrogen until it stops boiling (~10-15 sec),
9) Insert the tubes into a thermocycler with a program set for: 37°C for 5 minutes, 28°C for 1 hour.

Note: water baths or incubators at 37°C and 28°C can be used also.

10) Add 50 µL of liquid LB media and pipette onto 6-well agar plates with LB media containing the appropriate antibiotics for selection of the plasmid and Agrobacterium strain (for antibiotic working concentrations see below). Gently spread the liquid on the plate using orbital movements (Suppl. Video 1).
11) Culture the plates inverted at 28°C for 3 days.

Working concentrations of antibiotics for Agrobacterium selection

To a 500 ml bottle of media, add the appropriate combination of antibiotics:

1. 500 µL of a 50 mg/mL **kanamycin** (Melford, #K22000) stock in water (50 µg/mL final)
2. 250 µL of a 50 mg/mL **rifampicin** (Melford, #R64000) stock in DMSO (25 µg/mL final)
3. 500 µL of a 25 mg/mL **chloramphenicol** stock in ethanol (Sigma-Aldrich, #C0378) (25 µg/mL final)
4. 500 µL of a 25 mg/mL **gentamycin** (Duchefa, #1405-41-0) stock in water (25 µg/mL final)
5. 250 µL of a 100 mg/mL **spectinomycin** (Melford, #S23000) stock in water (50 µg/mL final)
