# Supplementary material for: Semi‐automated workflow for high‐throughput Agrobacterium‐mediated plant transformation
Source: Plant J. 2025 Apr 12;122(1):e70118. doi: 10.1111/tpj.70118 (PMC11993085; doi:10.1111/tpj.70118)
Supplement: Supplementary file 1 — Appendix S1. File S1. Protocol designer file for Agrobacterium competent cells dispensation in 96‐well plates. File S2. Protocol designer file for Agrobacterium transformation automation for 24 samples. File S3. Protocol designer file for Agrobacterium transformation automation for 96 samples transformation. File S4. Protocol designer file for plating Agrobacterium transformations. File S5. Jupyter Notebook protocol for Agrobacterium transformation. File S6. Jupyter Notebook protocol for plating Agrobacterium transformations. [file TPJ-122-0-s006.zip › Supplemental Document 2.pdf]

Supplemental Document 2.

Protocol for *Marchantia* transformation

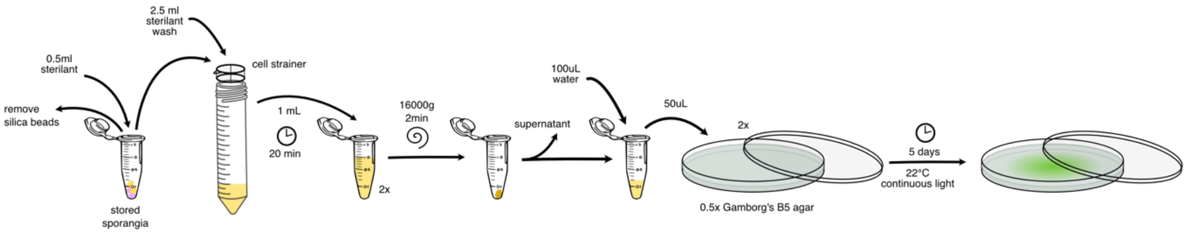

Figure 1. ***Steps for spore sterilization and preculture.***

1) Start from 1-2 archegoniophores dried in an Eppendorf tube with silica beads.
2) Remove the silica beads from the tube or move the archegoniophore to a new tube.
3) Prepare the sterilisation solution by adding one Milton mini sterilizing tablet (https://www.milton-tm.com/en/consumer/products/mini-sterilising-tablets) tablet to 10 ml of sterile water in a 50 mL Falcon tube. Allow to dissolve with mixing.
4) Add 200 μL of the sterilant solution in a tube with sporangia and crush and mix thoroughly with the tip to release the spores from the sporangia until the solution turns dark yellow-to-brown.
5) Add 300 μL of the sterilant solution and mix till homogeneous.
6) Place a 40 μM cell strainer (Greiner #542040) on a 50 mL Falcon tube and pour the 0.5 mL of the yellow liquid onto the filter avoiding green tissue to minimise clogging.
7) Wash the filter with an additional 2.5 mL of sterilant.
8) Divide the filtered solution containing spores (total volume ~3 mL) into two 1.5 mL Eppendorf tubes. Let the samples stand for 15-40 min.
9) Prepare two agar plates containing 0.5x Gamborg's B5 media.
10) Spin tubes at 16000 g for 2 min in a microcentrifuge.
11) Discard each supernatant without disturbing the yellow pellet, and pool spores in a single tube.
12) Re-suspend in ~100 μL sterile water and inoculate 50 μL on the 0.5x Gamborg's B5 agar plates and plate the spores using a sterile spreader.

Note: this quantity works well for ~24 transformations, adjust the amount depending on the number of transformations needed.

13) Seal the plates with micropore tape and culture for 5 days upside-down at 22 °C in continuous light. The inverted orientation prevents rhizoids entering the agar, and allows easy transfer of sporelings.

Agrobacterium and *Marchantia* co-culture

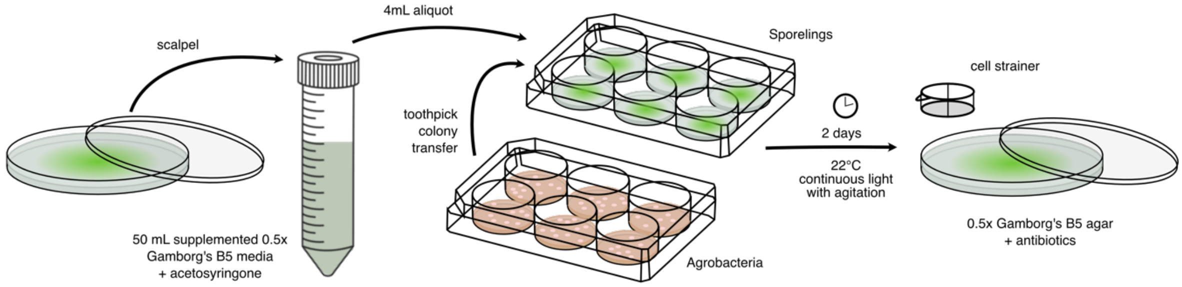

Figure 2. ***Marchantia co-culture with Agrobacteria and transformation.***

1) Collect sporelings from the two agar plates with a sterile scalpel and transfer them to two 50 mL Falcon tubes containing 50 mL of liquid 0.5x Gamborg's B5 media (plus supplements) and 100 μM acetosyringone (Phytotech #A104).

Note: adjust the volume of liquid media according to the number of planned transformations.

2) Add 4 mL to each well in four 6-well plates.
3) With a sterile tip or toothpick, scoop one colony of *Agrobacterium* with the appropriate plasmid and inoculate one well of sporelings (Suppl. Video 2).

Note: make sure that most of the bacteria does not remain in the tube or tip after inoculating. We recommend leaving one well without inoculation as a control for contamination. More than one Agrobacterium strain containing plasmids with different antibiotic resistance can be co-transformed. A 1-2 orders of magnitude reduction in efficiency should be expected. For that purpose, hygromycin, G418, and chlorosulfuron are the most effective antibiotics for Marchantia plant selection (Tsuboyama et al., 2018).

4) Repeat for each well.
5) Seal the 6-well plate with double micropore tape and grow for 2 days at 22°C with shaking and continuous light.

Note: co-culture should look cloudy but spores still green. If the co-culture is still clear after a day, repeat the *Agrobacterium* inoculation.

6) Using a wide-bore pipette, transfer the sporelings from each well to a 70 μm cell strainer (Greiner, #542070) in a 50 mL Falcon tube.
7) Wash the collected sporelings with 10 mL sterile water.
8) Using 400 μL of sterile water, wash the sporelings onto 60 mm Petri dish with 0.5x Gamborg's B5 agar media plus 100 μg/ml cefotaxime and selective antibiotic/s for the plasmid of interest (normally hygromycin).
9) Repeat for each well.
10) When the plate is dry, add double micropore tape and culture at 22°C with continuous light.

Note: the first clear signs of selective plant growth and (strongly expressed) visible genetic markers can be seen 5-7 days after culture.

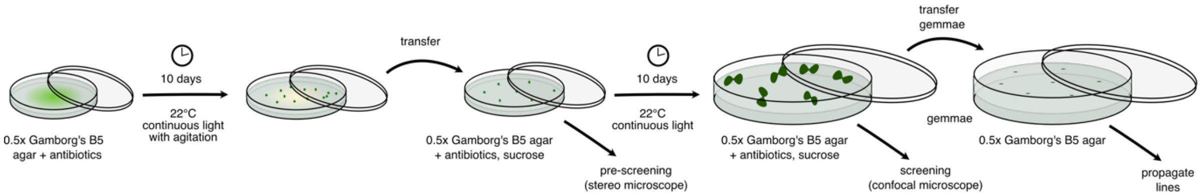

Figure 3. ***Selection of stable transgenic lines of Marchantia.***

11) After 10 days, using sterile tweezers, transfer 5-7 emerging thalli to a 90mm Petri dish with 0.5x Gamborg B5 agar with cefotaxime, selective antibiotic(s), and 0.5% (w/v) sucrose for a second round of selection, and culture in continuous light at 22°C.
12) When plants generate gemma cups (10-15 days later), transfer gemmae to a final plate without antibiotics. These gemma can be grown and imaged by microscopy for fluorescent reporters.

Media recipes

1L Supplemented Gamborg's B5 media for sporeling transformation (liquid co-culture media)

- 1.6 g Gamborg's B5 media (0.5x) (Phytotech, #G398)

- 20 g sucrose (2% w/v) (Fisher-Scientific S2-50GM)
- 0.30 g L-glutamine (0.03% w/v) (Sigma-Aldrich #G8540)
- 1g N-Z amine A (0.1% w/v) (Milipore #C0626)
- Add RO water to 1L
- Adjust pH to 5.7-5.8 with 1 M KOH

1L Gamborg's B5 media for *Marchantia* plates (solid)

- 1.6 g Gamborg's B5 media (0.5x) (Phytotech, #G398)
- 12 g Micropropagation-grade agar (Phytotech #A296)
- Add RO water to 1L
- Adjust pH to pH 5.7-5.8 with 1M KOH.

Note: for 2nd selection add 5 g sucrose (0.5% w/v)

Antibiotic working concentrations for plant culture

- 500 µL of a 100 mg/mL **cefotaxime** (Sigma-Aldrich, #64485-93-4) stock in water (100 µg/mL final)
- 200 µL of 50 mg/mL **hygromycin B** (ThermoFisher, #10687010) (20 µg/mL final)
- 50 µL of a 500 mM **chlorosulfuron** (Supelco, #34322) stock in DMSO (0.5µM final)
- 50 µL of 50 mg/mL **G418 sulfate** (Gibco, #10131035). (5 µg/mL final)
