# Supplementary material for: Semi‐automated workflow for high‐throughput Agrobacterium‐mediated plant transformation
Source: Plant J. 2025 Apr 12;122(1):e70118. doi: 10.1111/tpj.70118 (PMC11993085; doi:10.1111/tpj.70118)

**Supplemental Figure 1.** Layout of different protocols for automated *Agrobacterium* transformation protocols.


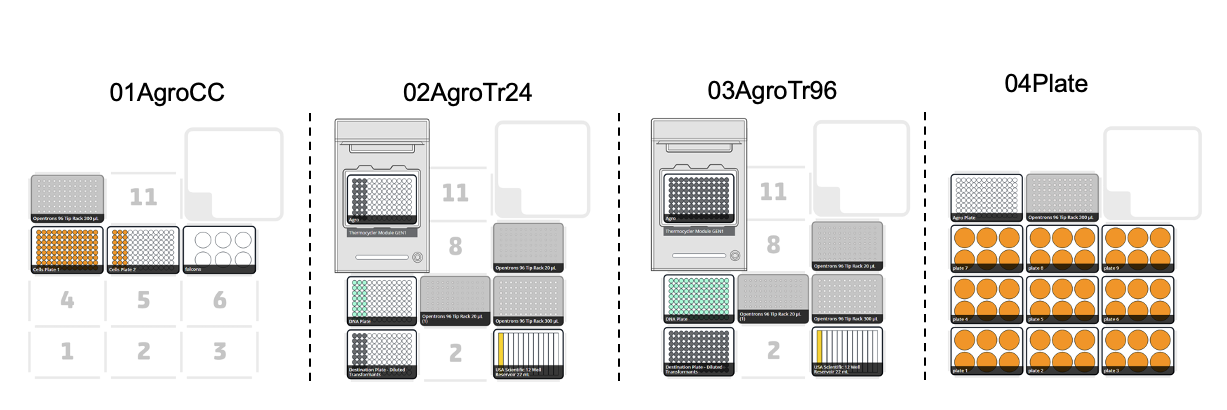

Supplement: Supplementary file 6 — Figure S1. Layout of different protocols for automated Agrobacterium transformation protocols. [file TPJ-122-0-s002.docx]
